# Prediction of secreted uncharacterized protein structures from *Beauveria bassiana* ARSEF 2860 unravels novel toxins-like families

**DOI:** 10.1038/s41598-025-02618-3

**Published:** 2025-05-22

**Authors:** Peter F. Farag, Aya A. Elsisi, Esraa W. Elabd, Jana J. Sadek, Nada H. Mousa, Rawan M. Zaky, Sara M. Ahmed

**Affiliations:** Department of Microbiology, Faculty of Science, Ain Shams University, Cairo, 11566 Egypt

**Keywords:** AlphaFold2, *Beauveria bassiana*, Insecticidal proteins, New families, Structural annotations, Microbiology, Structural biology

## Abstract

Insecticides are toxic substances used to control a wide variety of agricultural insect pests. Most of these are chemicals in nature, and their increasing residues in soil, water, and fruits contribute to environmental pollution, chronic human illnesses, and the emergence of insecticide resistance phenomenon. In the context of a green environment, bioinsecticide metabolites, including proteins, are a safe alternative that mostly has selective toxicity to insects. Thus, this study aimed to predict and identify new toxin-like families through uncharacterized secreted proteins from one of the most potent entomopathogenic fungi, *Beauveria bassiana* ARSEF 2860, which was selected as a model. In this work, a total of 2483 amino acid sequences of uncharacterized proteins (Ups) were retrieved from the RefSeq database. Among these, 365 UPs were identified as secreted proteins using the SignalP web server. We implemented the integration of well-designed bioinformatic tools to characterize and anticipate their homologous similarities at the sequence (InterPro) and structural (AlphaFold2) levels. The structural function annotation of these proteins was predicted using DeepFRI. With 269 successfully predicted folds, we identified new putative families with pathogenesis functions related to toxins like Janus-faced atracotoxins (insecticidal spider toxin), Cry toxins (commercial insecticide from *Bacillus thuringiensis*), ARTs-like toxins, and other insecticidal toxins. Furthermore, some proteins that are not homologous to any known experimental data were functionally predicted as cation metal ion binding (Zn, Na, and Co) with potential toxicity. Collectively, computational structural genomics can be used to study host–pathogen interactions and predict novel families.

## Introduction

*Beauveria bassiana* (Balsamo-Crivelli) Vuillemin is one of the most devastating necrotrophic soil-borne entomopathogenic fungus that belongs to the Cordycipitaceae family^1,2^. It causes white muscardine disease in more than 700 insects and spider mite species across 15 orders and 149 families^3,4^. This fungus is characterized by secreting several natural selective pigments and toxins (beauvericin, bassiana, bassianolide, tenellin, beauverolides, oosporein, and so forth) with highly virulent effect, making it commercially manufactured as an eco-friendly mycoinsecticide and used in Integrated Pest Management (IPM) programs^5,6^. *B. bassiana* ARSEF 2860 is a popular strain for pest control^7^. It was isolated from *Schizaphis graminum* (wheat aphid), and its genome (GenBank accession number ASM28067v1) was recently assembled^8,9^. This enables the study of various genes that encode insecticidal proteins, illuminating the mechanism of action and their virulence against insects.

Many proteins encoded by most microorganisms are known to lack experimental proof of translation for their in vivo expression. These proteins, which constitute between 20 and 50% of the protein-coding regions, are referred to as hypothetical proteins (HPs), and their roles remain unclear^10–12^. HPs can be categorized as uncharacterized proteins (UPs) and the domain of unknown functions (DUF)^13^. Despite the experimental confirmation of their existence, UPs have not yet been named or linked to a known gene. In contrast, DUFs are proteins identified through experiments but do not have recognized structural or functional domains^14^. Most of these proteins are expected to play crucial roles, and their annotation may uncover new domains and motifs, functional pathways, and discoveries of novel pathogenesis-related genes with putative toxin-like family homology^15,16^.

While many obstacles remain in annotating these types of proteins, numerous bioinformatics tools, including databases and web servers, are available for functional annotation and homology assignment for UPs^16,17^. Despite the widespread popularity of sequence-based annotation tools like BlastP and InterPro, many protein sequences remain unclassified and functionally unannotated^18^. This could be attributed to the rapid divergence between homologous proteins, leading to diminished sequence similarity^19^. Structure-based annotation depends on building a protein’s three-dimensional (3D) structure, which reveals molecular functions, novel folds, and structural similarities, resulting in enhanced genomic annotations^20,21^. Experimental structure determination is a costly and time-consuming process, making computational structure prediction an appealing alternative for reducing the effort needed to obtain a structural model from months of laboratory work to just a few keystrokes^22,23^.

The AlphaFold Protein Structure Database (AFDB) is a publicly accessible data collection of protein structures and their confidence metrics (pLDDT 0–100), generated by the AlphaFold2 (AF2) artificial intelligence system^24,25^. Recently, an AlphaFold 3 (AF3) webserver was launched to predict structures molecular interactions^26^. The homologous structural similarity of proteins can be measured using predicted template modeling (pTM) scores, which range from 0 to 1. A TM-align score greater than 0.5 indicates the evolutionary relatedness between two structures adopting the same fold. Thus, structural comparison can reveal this startling similarity, which remains elusive for BLAST and other sequence-based methods such as HHblits^27^.

Considering the significance of studying the fungal secretome (the proteins secreted outside the plasma membrane) that play a vital role in host–pathogen interactions, knowledge about these proteins remains limited in many fungal species^28–31^. So, the purpose of this study is to describe the secreted UPs encoded by *Beauveria bassiana* ARSF 2680, aiming to explore potential novel toxin-like families based on structural annotations that enhance the understanding of the mechanism of action. This will be achieved by constructing 3D structures for these proteins and then utilizing the homology model to predict their functions (structure-based functions) as well as paralogue and orthologue similarities.

## Materials and methods

### Sequence information and retrieval

The information data of *Beauveria bassiana* ARSEF 2860 (Genbank accession no. ADAH00000000.1) was submitted to the NCBI database by Zhejiang University, China^9^. The genome of this strain has a length of 33.7 Mb and contains 10,364 genes encoding 10,364 proteins. Out of these, 2483 (24%) of the proteins were classified as UPs, whereas 7881 (76%) were fully characterized proteins. The proteome of this strain was retrieved from the NCBI RefSeq database (https://www.ncbi.nlm.nih.gov/datasets/gene/GCF_000280675.1/, accessed on 25 August 2024) that was submitted recently in January 2024.

### Screening of secreted proteins

Secreted proteins that carry a signal peptide (SP) were predicted using SignalP v5.0^32^. DeepTMHMM V1.0.24 and TMHMM 2.0 web servers were used to detect the transmembrane helix proteins^33^. The candidates were excluded if they contained any transmembrane helices.

### Domain prediction, homologous similarity, and clustering

The domains of 365-secreted protein sequences were predicted by InterPro 98^34^. Based on structure prediction, the domains were first screened through AFDB v2 and then re-predicted by AF2 using ColabFold v1.5.5 implementation after removing the SP sequence^35^. Five models were created for every protein, and we selected the top model based on its best average pLDDT score. RUPEE^36,37^ was used for searching homologous structural similarity against SCOPe v2.08, CATH v4.3, and PDB chain databases downloaded on 16 July 2022 (Top aligned, Full length), while BlastP was used for sequence similarity searches. Two structures were deemed similar if their TM scores exceeded 0.5. A similar network was established based on structural similarity through all-against-all comparisons using the DALI server^38^. Cytoscape v3.10 was employed to construct and visualize the protein network, and ChimeraX v1.6.1 was used for visualizing 3D protein structures^39,40^.

### Prediction of protein functions

The putative functional annotation to secreted UPs was performed using a DeepFRI (cut-off score ≥ 0.5) to predict enriched Gene Ontology (GO) terms of biological process (BP) and molecular function (MF) based on protein structures^41^. Argot^2.5^ web server (cut-off score > 200) was used to predict enriched GO terms according to protein sequences^42^, where these sequences were downloaded from the NCBI database in FASTA format. ToxinPred 2.0 (cut-off score > 0.2) was used for the anticipated toxicity of some sequences^43^.

### Biomolecular interactions and molecular docking analysis

The interactions between a subset of UPs and other molecules, including ions, nucleic acids, small molecules, and modified residues, were predicted using the AF3 server^26^. Protein–ligand docking was performed using CB-Dock2^44^, whilst protein–protein docking was conducted between the receptor and target proteins using the HDOCK server^45^.

## Results and discussion

### Statistical insight into describing the uncharacterized secreted proteins

**Fig. 1:**
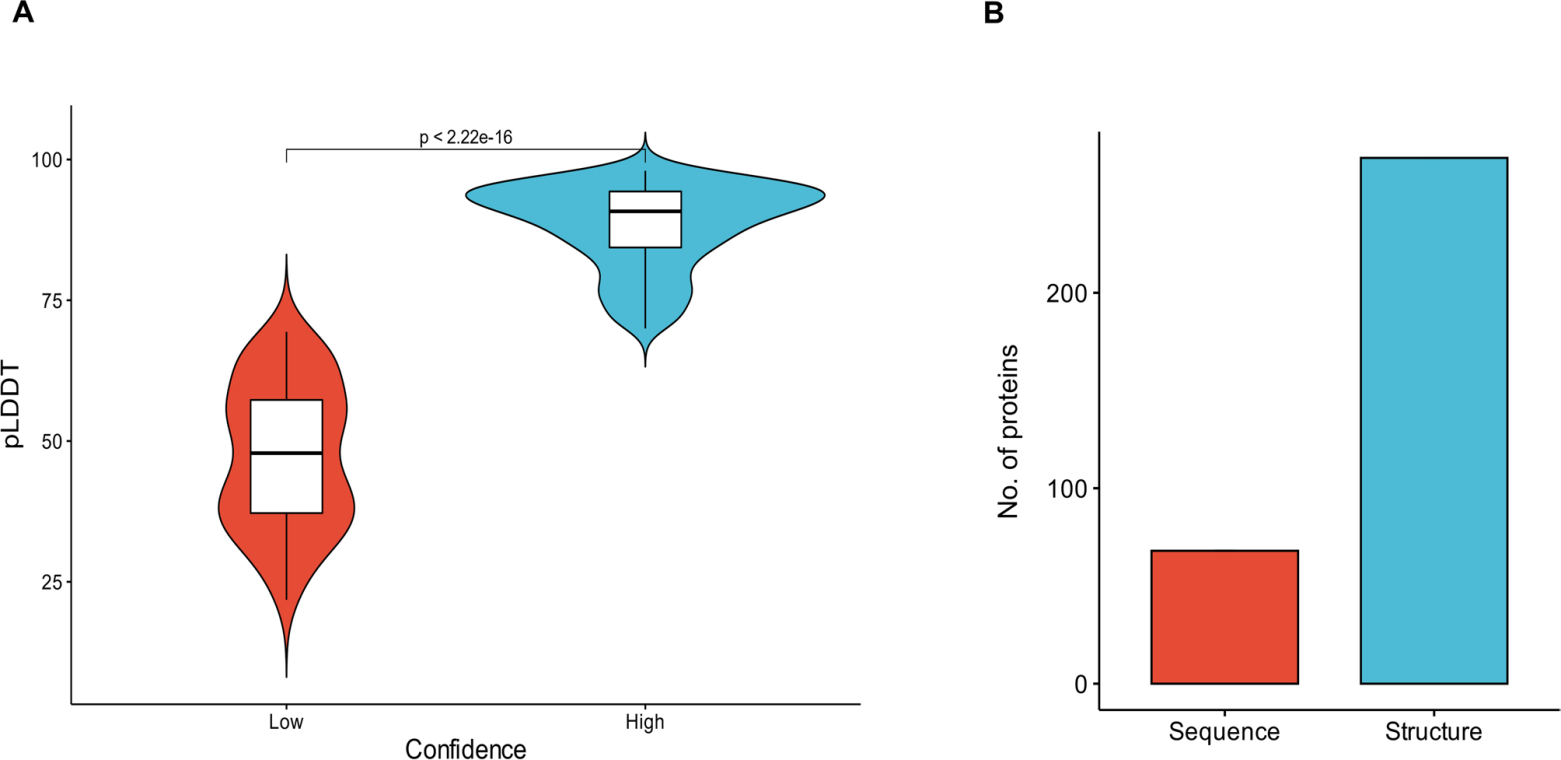
(**A**) The violin box plot showed successful screening for folds with high confidence scores (pLDDT > 70). (**B**) The bar plot showed the comparison between the number of annotated proteins by InterPro (sequence-based annotation, n = 68) and AF2 (structure-based annotation, n = 269).

In this study, we used sequence and structure predictions on the putatively secreted UPs (n = 365). The structural folds of 269 proteins (74%) were predicted using AF2 with high confidence scores (pLDDT > 70), resulting in a significant value (*p* < 0.0001) compared to projected low confidence scores (Fig. 1A, Supplementary Table S1). Only 68 proteins could be predicted from primary sequences, with more than 80% lacking InterPro annotation (Fig. 1B). Additionally, most of the detected results involved in this study were annotated according to the structure of these proteins. These results are not surprising; other recent research utilized structural annotations to identify novel families through AI machine-learning modeling^18,19,22,25^. Using Argot2.5 and DeepFRI for functional annotation, most toxin-like clusters exhibit a putative pathogenesis biological process (GO:0009405), highlighting the potential role of these proteins in the fungus’ pathogenicity against its host. To reveal the different putative toxin families, the high pLDDT-predicted UP structures were clustered based on a structural alignment against other toxin proteins with known structures. Finally, we summarized the various protein clusters as follows:

### Identification of predicted toxin-like proteins with a knottin fold

We investigated structural similarity within two distinct clusters, including the knottin fold. We discovered that these clusters might represent new families: the first resembles a spider toxin family, while the second is similar to bubble proteins. Knottins, or inhibitor cystine knots (ICKs), are structural cysteine-rich protein families (30–50 amino acids) that are classified in the SCOPe database^46,47^. This family is found in many living organisms, including the toxins of venomous animals like spiders and scorpions. Additionally, it features a distinctive knotted structure formed by three intramolecular disulfide linkages, which offer great chemical, thermal, and proteolytic stability^48^.

**Fig. 2:**
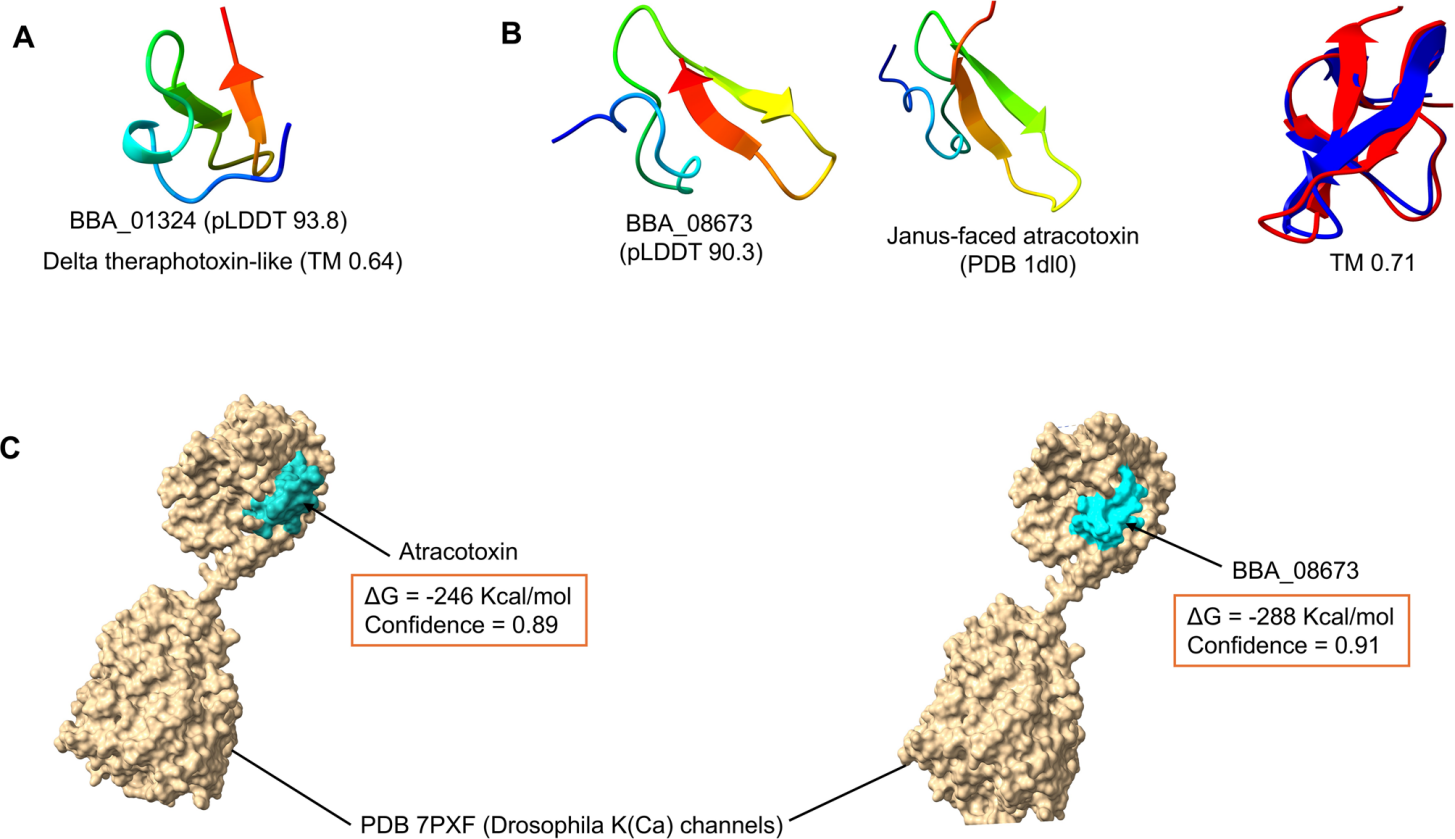
New spider toxin-like families. (**A**) delta-theraphotoxin-like protein (BBA_01324) with a TM score equal to 0.64. (**B**) J-ACTX-like protein (BBA_08673) superposed to Atracotoxin PDB 1dl0 (TM 0.71). (**C**) Docked complex between J-ACTX and BBA_08673 against *Drosophila* K (Ca) channel.

After matching with the SCOPe fold database v2.08, we discovered several genes, including BBA_06834, BBA_01324, BBA_03436, BBA_08673, BBA_09303, and BBA_09080, that encoded proteins similar to spider toxin structures. Spider toxin proteins belong to a diverse family of knottins that include various insecticidal peptides targeting neuronal ion channels and receptors. The outstanding specificity, efficacy, and stability of these peptides have attracted significant interest as potential eco-friendly insecticides^49^. From these genes, BBA_01324 (pLDDT 93.8) was similar to delta-theraphotoxin with a TM score of about 0.65. This gene is species-specific to *Beauveria* based on sequence (BLASTp) and structure (AFDB) levels (Fig. 2A). Moreover, we investigated a vital gene (BBA_08673), exclusive only to this strain, similar to insect-selective neurotoxin Janus-faced atracotoxins (J-ACTXs) from the venom of the Australian funnel-web spider (*Hadronyche versuta*) that seems to be a promising target for insects^50^. Figure 2B demonstrates a very high confidence score (pLDDT 90.3) of this protein structure and the superposed score (TM 0.71), indicating an acceptable similarity to J-ACTX (PDB 1DL0). This toxin is a specific blocker of insect K(Ca) channels^51^. Thus, the molecular docking analysis between the original toxin and our protein against the *Drosophila* K (ca) channel (PDB 7PXF) indicated a similar active site and binding affinity (Fig. 2C). While J-ACTX shares a disulfide connection pattern akin to other ICKs, it exhibits limited sequence homology to any protein and DNA sequence databases^52^. The structural similarity between the fungal and spider toxins gives the fungus an edge in mass production for a scalable industry.

**Fig. 3:**
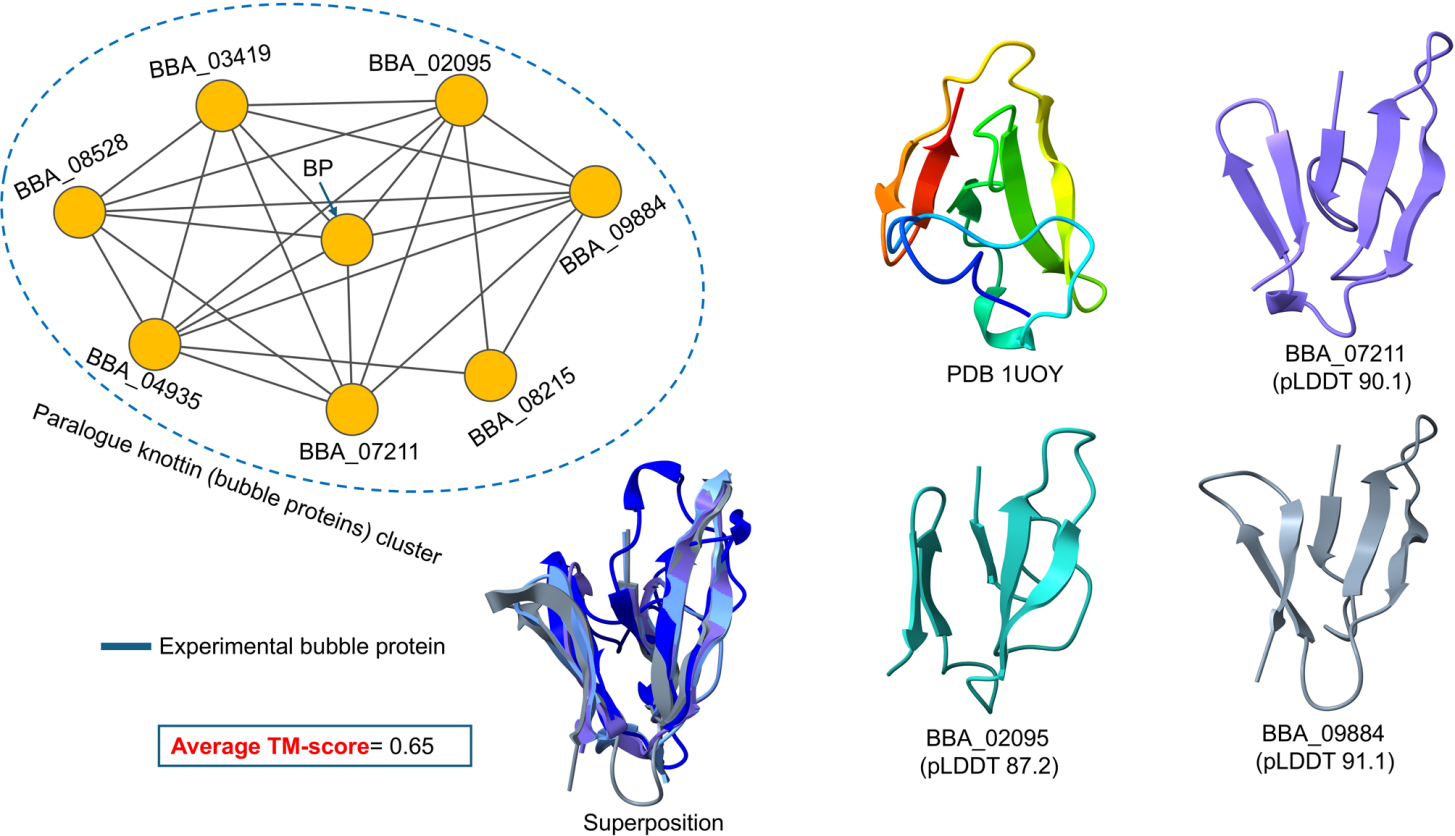
A cluster of seven BP-like and three high-confidence score models with an average alignment score of 0.65 compared to experimental bubble protein (PDB 1UOY). Grey edges represent structural similarities among these proteins.

Another knottin family had similarities to bubble protein (BP), which was initially identified in *Penicillium brevicompactum* exudate and may act as a toxin against fungi^53^. This cluster contains seven BP-like proteins, was the average TM-score of representatives aligned with the experimental BP (PDB 1UOY) being 0.65, as illustrated in Fig. 3. These proteins are structurally similar to other antifungal proteins, including the *P. chrysogenum* antifungal protein (PAF) family. BP is categorized as a member of defensins, which consist of five beta sheets and structural classification places BP into knottin fold-containing proteins^54^. Thus, this investigation suggests that *B. bassiana* might be a good biopesticide against insects and fungi.

### Clustering of Cry toxin-like proteins

**Fig. 4:**
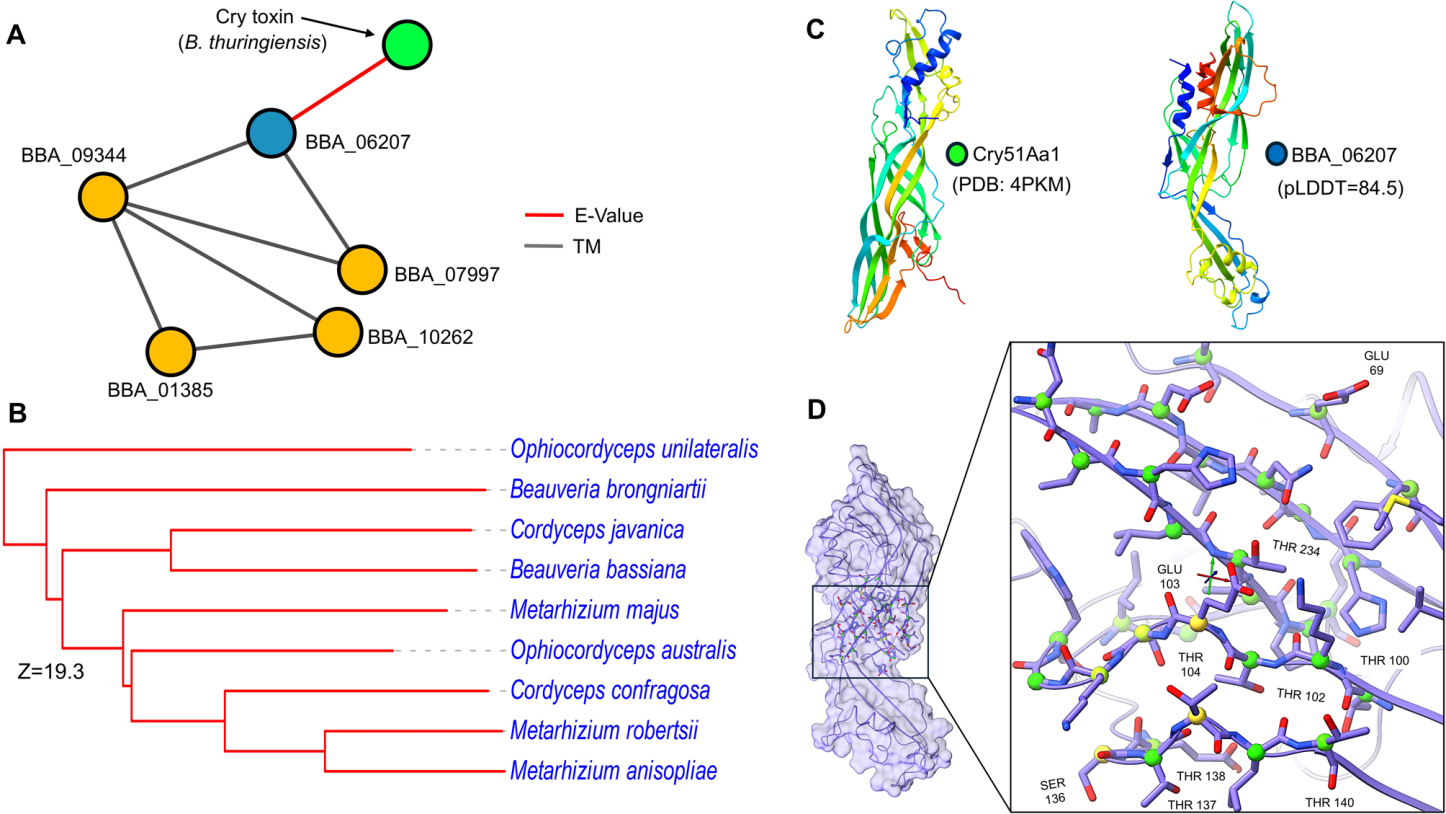
Putative new Cry toxin-like family based on structural homology. (**A**) The network of members of *B. bassiana* compared to Bt Cry toxin Cry51Aa1. Each orange node is similar to each other by calculating TM-score > 0.7 (Grey edge). In contrast, the green node represents the BBA_06207 protein (Blue node) with a high E-value (Red edge) structural similarity with Bt Cry toxin (Green node). (**B**) Structural similarity dendrogram between highly similar protein from other entomopathogenic fungi to BBA_09344 protein. The average DALI Z-score was 19.3 (cutoff Z = 2). (**C**) 3D structure comparison between Cry51Aa1 (PDB: 4PKM) and BBA_06207 (confidence score = 84.5). (**D**) Active site of BBA_06207 with threonine-rich residues.

With structure-based clustering, we were able to capture a new family specific to entomopathogenic fungi that are related to *Bacillus thuringiensis* (Bt) Cry toxins (Fig. 4). Crystal (Cry) proteins are selective, pore-forming toxins that specially target midgut invertebrates and are generally innocuous to mammals. They are widely employed as agricultural pesticides to eliminate insects and nematodes^55^. A cluster of five proteins encoded by BBA_06207, BBA_01385, BBA_09344, BBA_10262, and BBA_07997 genes in *B. bassiana* exhibited varied degrees of resemblance to Cry51Aa1 Cry toxin, an insecticidal aerolysin-type β-pore-forming toxin composed of 309 amino acid sequences^56^. Compared with the members in this cluster, BBA_06207 from *B. bassiana* exhibits higher folding similarity (E-value < 10^−7^) than other proteins from the same fungus or other entomopathogenic fungi. Concurrently, BBA_09344, BBA_07997, BBA_10262, and BBA_01385 have structural alignment with TM prediction scores greater than 0.7 (Fig. 4A). The BBA_09344 gene exhibited the most common fold sharing among other genes, making it suitable for clustering with proteins identified in other entomopathogenic fungi through AFDB clusters and aligned using the DALI server, resulting in Z-score outputs. The dendrogram is generated by average linkage clustering of the structural similarity matrix (DALI Z-scores), with a similarity cutoff at Z = 2^57^. The results reveal a structural similarity dendrogram between them, giving a high Z DALI score (Z = 19.3) and a strong similarity between a query protein in *B. bassiana* and *Cordyceps javanica* protein (IF1G_09556) (Fig. 4B). Figure 4C,D illustrate the putative 3D structure and active site of BBA_06207, a likeness to Cry51Aa1, which is rich with threonine residues in the middle of the backbone. In previous works, serine or threonine residues were shown to make up about 23% of the different types of Cry toxins, including Cry51Aal^56,58^.

### Putative insecticidal GNIP1Aa toxin-like proteins

**Fig. 5:**
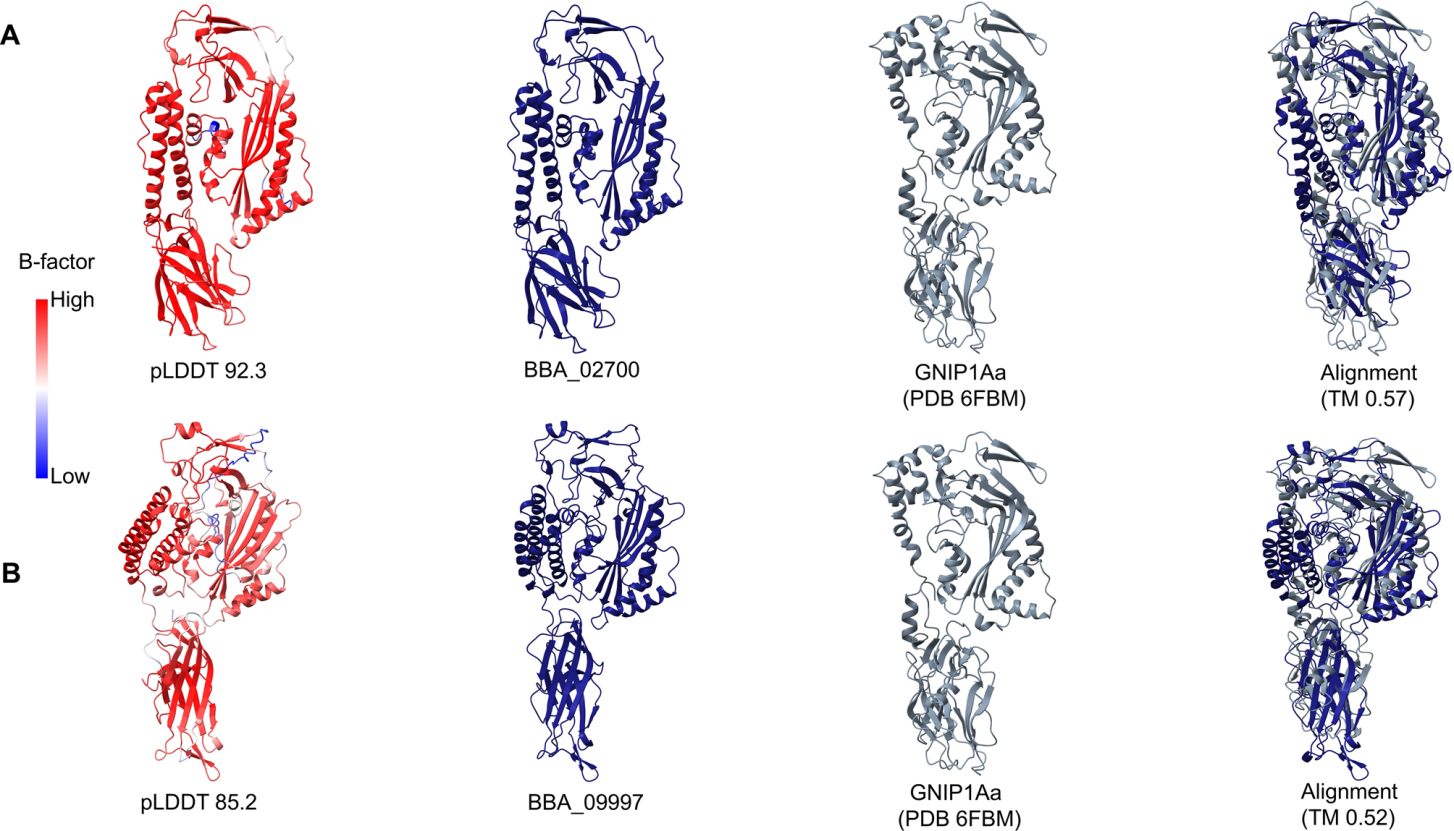
Structural superposition between the predicted and solved structures. (**A**) The structure of BBA_02700 protein predicted by AF2 was superposed with TM-align against the experimental structure (PDB: 6FBM). (**B**) The structure of BBA_09997 protein was superposed with TM-align against 6FBM.

We identified two proteins (BBA_02700 and BBA_09997) that exhibit structural similarities to the insecticidal GNIP1Aa protein, which belongs to the membrane attack complex/PerForin (MACPF) family. BBA_02700 demonstrated a very high confidence score (pLDDT 92.3) and was superposed onto GNIP1Aa (PDB: 6FBM) with a TM value of 0.57 (E-value < 10^−5^) (Fig. 5A). Also, BBA_09997 presented a high confidence score (pLDDT 85.2) and was aligned onto GNIP1Aa (PDB: 6FBM) with a TM value of 0.52 (E-value < 10^−5^) (Fig. 5B). GNIP1Aa is a protein identified from *Chromobacterium piscinae* in 2017 that displays particular toxicity against Western corn rootworm (WCR), one of the most destructive corn pests in the United States. Although GNIP1Aa belongs to the same class of Cry toxins, it is distinct from all insect-control treatments currently available on the market that utilize modern agricultural technologies. Due to its distinctiveness and protein activity, GNIP1Aa is a strong commercial candidate for development into a transgenic product. Such a solution would be highly effective in preventing crop loss in corn and delaying the emergence of pest resistance^59,60^. Other entomopathogenic fungi, including *Cordyceps javanica* (Gene: IF1G_04403), *Ophiocordyceps camponoti-leonard* (Gene: CP532_0387), and *Metarhizium anisopliae* (Gene: MAN_10237) have comparable structures following structure-based grouping.

### Clustering of ARTs-like toxins

**Fig. 6:**
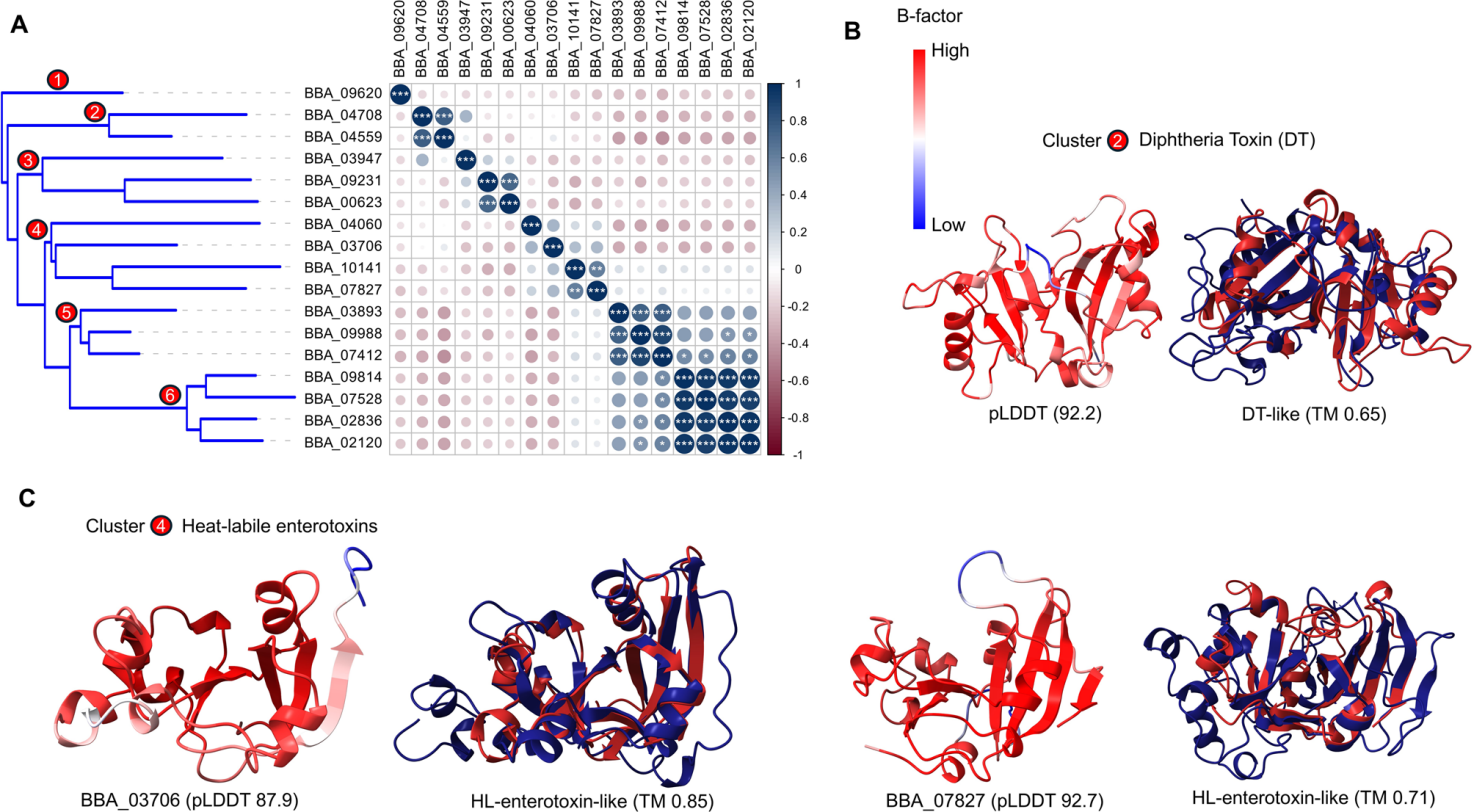
Structural similarity analysis of ADP-ribosylation fold-containing proteins. (**A**) Dendrogram and similarity matrix of 17 clustering members. (**B**) Homology of Cluster 2 to diphtheria toxin. (**C**) Homology of Cluster 4 to heat-labile enterotoxin. *P < 0.05, **P < 0.01, and ***P < 0.001.

One of the largest clusters, including 17 members, was described with ADP-ribosylation fold and NAD^+^-dependent ADP-ribosyltransferase activity according to MF (GO:0003950); six of them possessed predicted structures with estimated TM > 0.6 matching known homologous proteins (Fig. 6). Catalysis of these proteins evolved by a structural superfamily of enzymes, called ADP-ribosyltransferases (ARTs) with NAD^+^ as a co-substrate^61^. The paralogue distribution of these proteins was clustered into five groups and one singleton as shown in Fig. 6A, where the orthologue similarity of these groups was exclusive only to entomopathogenic fungi, especially *Metarhizium* and *Cordyceps* species, based on the sequence and structure homology clustering. Cluster 2 contained two proteins (BBA_04708 and BBA_04559) similar to diphtheria toxin (DT), a secreted exotoxin by *Corynebacterium diphtheriae*, with a high confidence score (pLDDT 92.2) and TM-align score about 0.65 (Fig. 6B). Figure 6C illustrated representative of two protein structures (BBA_03706 and BBA_07827) from Cluster 4, which analyzed for structural similarity to the heat-labile enterotoxin. The structure of BBA_03706 was determined with high confidence (pLDDT = 87.9), and structural alignment with the heat-labile enterotoxin revealed high structural similarity with a TM-score of 0.85. Similarly, the structure of BBA_07827 was determined with very high confidence (pLDDT = 92.7), and alignment with the heat-labile enterotoxin also showed high similarity, with a TM-score of 0.71. Historically, various classes of enzymes form the ART superfamily, including the diphtheria-toxin-like transferases (ARTDs) and the cholera-toxin-like transferases (ARTC)^62^. Although it is uncommon for secreting toxins to resemble human-infecting bacterial toxins from entomopathogenic fungi, Aravind et al.^63^ reported the putative expanded evolution of ARTs throughout eukaryotes by horizontal gene transfer.

### Discovery of potential novel families with putative toxicity

**Fig. 7:**
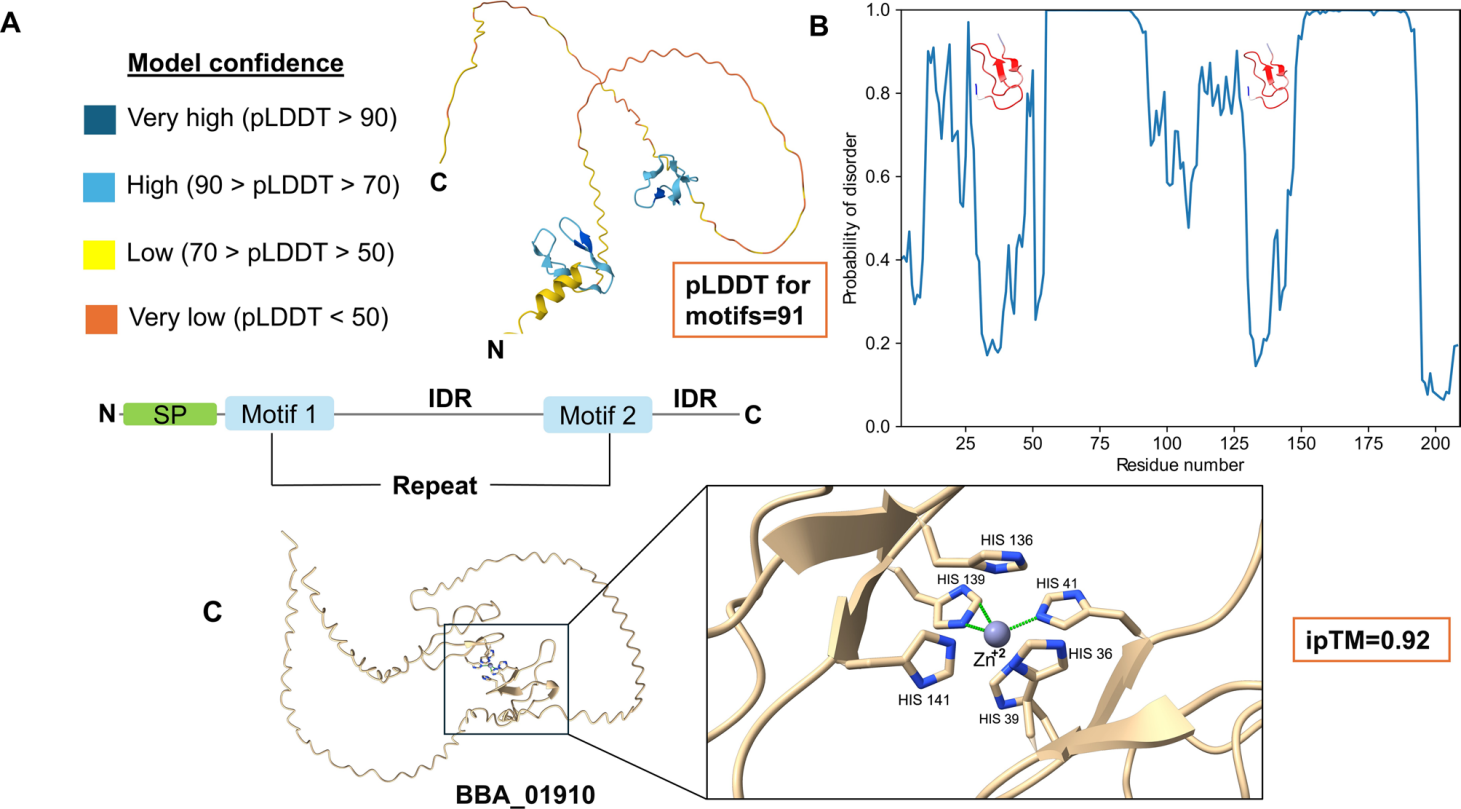
Characterization of BBA_01910 protein structure. (**A**) Prediction of the protein and folding of its repeated motifs with a confidence scale. (**B**) ODINPred was used to predict the intrinsically disordered regions for the protein (cutoff > 0.5). (**C**) Prediction of the binding sites between the BBA_01910 protein and zinc ion using AF3.

**Fig. 8:**
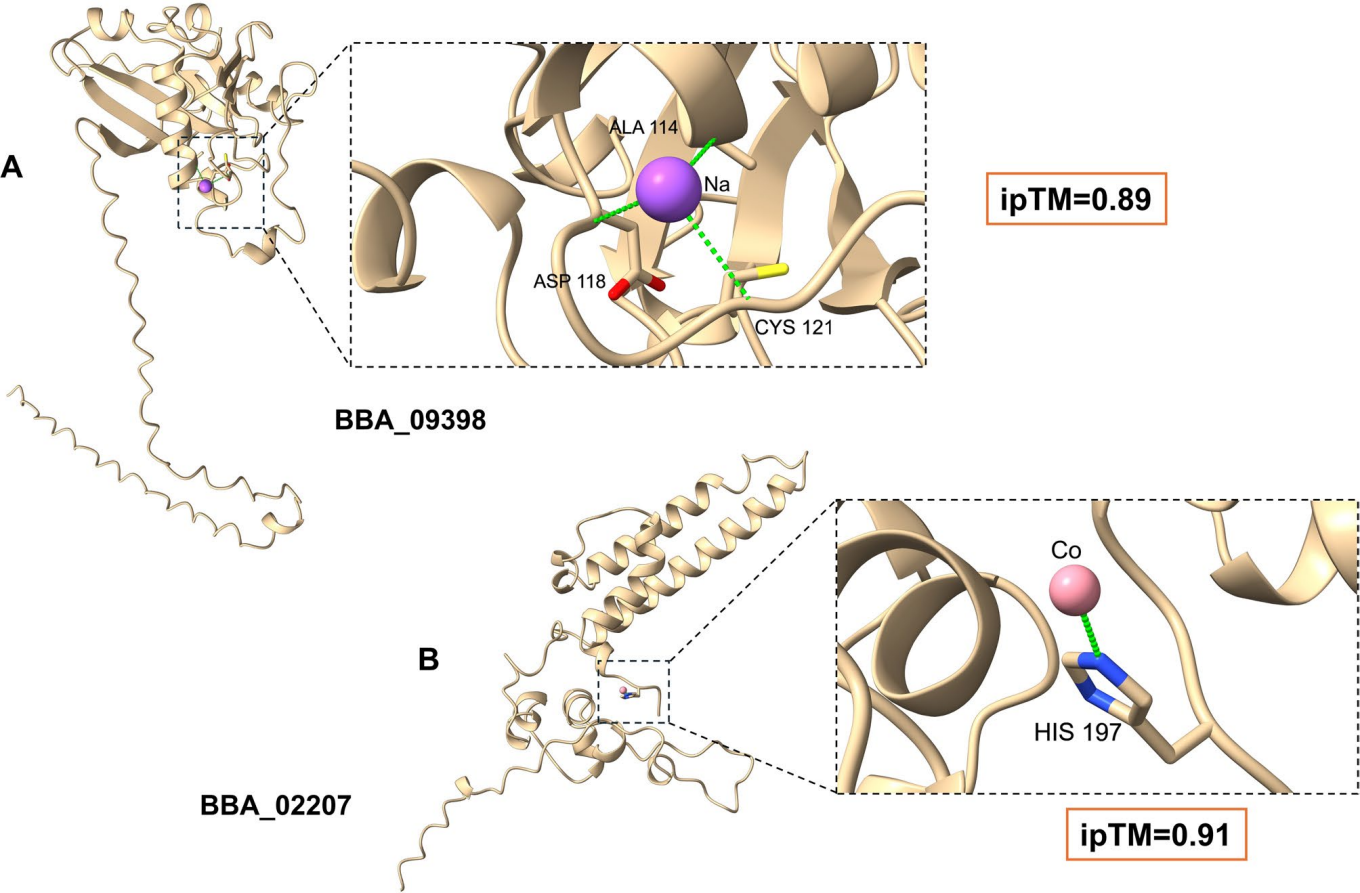
The interactions between (**A**) the BBA_09398 protein and sodium ion. (**B**) the BBA_02207 protein and cobalt ion.

Several proteins were identified with no homology to any known experimental proteins (TM < 0.4), forming novel families with putative MFs and biological processes. The selection criteria were critical as they concentrated on anticipated toxicity using ToxinPred 2.0 and were restricted to the entomopathogenic fungus group. Furthermore, a set of proteins appeared to share the same MF: cation metal ion binding (GO:0043169). From these proteins, BBA_01910 (209 amino acids) contains two repeated motifs fused with intrinsically disordered regions (IDRs) detected by the ODINPred server^64^, in which proteins with IDRs are noted to be highly prevalent in diseases^65^ (Fig. 7A,B). ToxinPred 2.0 predicted the toxicity of the motif sequence (ATCEPHEDHWHCPAGVPQPSLNPDGTPNPKATQ) with a score of approximately 0.75. To predict the type of cation metal ions, AlphaFold3 (AF3) was utilized for biomolecular interaction detection between the query protein and various ions, with results provided by interface-predicted template modeling (ipTM) scores^26^. Zinc ion interacted with six histidine residues (His-tag) and was the best matching ion to the studied protein giving a high ipTM score equal to 0.92 (Fig. 7C). Other novel folds were detected from genes BBA_09398 (pLDDT 85.4) and BBA_02207 (pLDDT 80.2) (Fig. 8). BBA_09398 (236 a.a.) binds to sodium ion through three residues (alanine, asparagine, and cysteine) with a high ipTM score (0.89) (Fig. 8A), while BBA_02207 (215 a.a.) attaches with cobalt ion through histidine residue (ipTM 0.91) (Fig. 8B). Like humans and plants, insects depend on various metal ions such as zinc, sodium, and calcium for proper physiological functions, where chelating these ions by any compound may block their functions^66^. Zinc metal is a necessary cofactor for many enzymes and is involved in a variety of processes, such as DNA synthesis, oxidation reactions, and cuticle production^67^. In neurons and other excitable cells, sodium ions are necessary for the propagation of the action potential. Numerous synthetic and naturally occurring neurotoxins, including various types of insecticides, target sodium channels due to their crucial functions in electrical signaling^68^. Cobalt has a key role in the synthesis of hemoglobin, which is necessary for insects to transport oxygen, as well as the metabolism of lipids, carbohydrates, and amino acids^69^.

In conclusion, the prediction of secreted uncharacterized protein structures from *Beauveria bassiana* ARSEF 2860 uncovered: (i) new pathogenesis-related proteins belong to putative toxin-like families, most of which exhibit potential pesticides for controlling insects and fungi, (ii) the evolution of expanded putative ADP-ribose transferases (ARTs-like family), (iii) mechanisms and functions of nonhomologous novel folds.

## Limitations and future perspectives

While computational structural genomics has proven to be an excellent supplement to the costly and time-consuming wet lab setting, some limits in our work must be acknowledged. Firstly, AF2 was unable to predict approximately 25% of protein structures. Secondly, several proteins did not fit with any known annotation category. Lastly, the structural prediction is insufficient to predict the putative functions. Despite these limitations, in silico structural-based annotation is a first step toward future studies that will focus on in vitro and in vivo validations of such proteins to assess their insecticidal efficacy and potential for agricultural uses. Regarding the non-homology protein structures, future advances might lead to their complete annotation, and these structures might be useful for the scientific community.

## Supplementary Information

Supplementary Information.

## Data Availability

The datasets generated and/or analysed during the current study are available in the NCBI RefSeq database under the Bioproject accession number PRJNA225503.

## Abbreviations

AF2: AlphaFold2
AF3: AlphaFold3
AFDB: AlphaFold database
ART: ADP-ribotransferase
BP: Bubble protein
GO: Gene ontology
pLDDT: Predicted local distance difference test
pTM: Predicted template modeling
SP: Signal peptide
UPs: Uncharacterized proteins
